# Supplementary material for: HIVprotI: an integrated web based platform for prediction and design of HIV proteins inhibitors
Source: J Cheminform. 2018 Mar 9;10:12. doi: 10.1186/s13321-018-0266-y (PMC5845081; doi:10.1186/s13321-018-0266-y)
Supplement: Supplementary file 2 — Additional file 2. Source code of HIVProtI web server. [file 13321_2018_266_MOESM2_ESM.zip › HIVprotI_Source-code/hivprotI/weka-3-6-6/doc/weka/classifiers/bayes/blr/GaussianPriorImpl.html]

GaussianPriorImpl


---


|  |  |  |
| --- | --- | --- |
| **Overview** \| **Package** \| **Class** \| **Tree** \| **Deprecated** \| **Index** \| **Help** \| **Weka's home** | |  |
| PREV CLASS   **NEXT CLASS** | **FRAMES**    **NO FRAMES**     **All Classes** |  |
| SUMMARY: NESTED \| FIELD \| CONSTR \| METHOD | DETAIL: FIELD \| CONSTR \| METHOD |  |


---


## weka.classifiers.bayes.blr Class GaussianPriorImpl

```
java.lang.Object
  weka.classifiers.bayes.blr.Prior
      weka.classifiers.bayes.blr.GaussianPriorImpl
```

**All Implemented Interfaces:**: java.io.Serializable, RevisionHandler

---

``` public class GaussianPriorImpl extends Prior ```

Implementation of the Gaussian Prior update function based on
CLG Algorithm with a certain Trust Region Update.
The values are updated in the BayesianLogisticRegressionV variables
used by the algorithm.

**Version:**
:   $Revision: 1.2 $

**Author:**
:   Navendu Garg(gargnav@iit.edu)

**See Also:**: Serialized Form

---

**Constructor Summary**

`GaussianPriorImpl()`


| **Method Summary** | |
| --- | --- |
| `void` | `computeLoglikelihood(double[] betas, Instances instances)`             This method calls the log-likelihood implemented in the Prior abstract class. |
| `void` | `computePenalty(double[] betas, double[] hyperparameters)`             This function computes the penalty term specific to Gaussian distribution. |
| `java.lang.String` | `getRevision()`             Returns the revision string. |
| `double` | `update(int j, Instances instances, double beta, double hyperparameter, double[] r, double deltaV)`             Update function specific to Laplace Prior. |

**Methods inherited from class weka.classifiers.bayes.blr.Prior**

`computelogLikelihood, getLoglikelihood, getLogPosterior, getPenalty`

**Methods inherited from class java.lang.Object**

`equals, getClass, hashCode, notify, notifyAll, toString, wait, wait, wait`

**Constructor Detail**

### GaussianPriorImpl

```
public GaussianPriorImpl()
```


**Method Detail**

### update

```
public double update(int j,
                     Instances instances,
                     double beta,
                     double hyperparameter,
                     double[] r,
                     double deltaV)
```

:   Update function specific to Laplace Prior.

    :   **Overrides:**: `update` in class `Prior`

---


### computeLoglikelihood

```
public void computeLoglikelihood(double[] betas,
                                 Instances instances)
```

:   This method calls the log-likelihood implemented in the Prior
    abstract class.

    :   **Parameters:**: `betas` -: `instances` -

---


### computePenalty

```
public void computePenalty(double[] betas,
                           double[] hyperparameters)
```

:   This function computes the penalty term specific to Gaussian distribution.

    :   **Overrides:**: `computePenalty` in class `Prior`
    :   **Parameters:**: `betas` -: `hyperparameters` -

---


### getRevision

```
public java.lang.String getRevision()
```

:   Returns the revision string.

    :   **Returns:**: the revision


---


|  |  |  |
| --- | --- | --- |
| **Overview** \| **Package** \| **Class** \| **Tree** \| **Deprecated** \| **Index** \| **Help** \| **Weka's home** | |  |
| PREV CLASS   **NEXT CLASS** | **FRAMES**    **NO FRAMES**     **All Classes** |  |
| SUMMARY: NESTED \| FIELD \| CONSTR \| METHOD | DETAIL: FIELD \| CONSTR \| METHOD |  |


---
